# Looking for idiopathic intracranial hypertension in patients with chronic fatigue syndrome

**Published:** 2013-04

**Authors:** Nicholas Higgins, John Pickard, Andrew Lever

**Affiliations:** 1 Addenbrooke’s Hospital, Cambridge CB2 0QQ; 2 Addenbrooke’s Hospital, Cambridge CB2 0QQ; 3 Addenbrooke’s Hospital, Cambridge CB2 0QQ

**Keywords:** Chronic fatigue syndrome, idiopathic intracranial hypertension, headache, lumbar puncture

## Abstract

**Introduction:**

Headache is common in chronic fatigue syndrome, a condition of unknown cause in which there are no clinical signs. Fatigue is common in idiopathic intracranial hypertension, a headache condition of unknown cause in which the only clinical signs are those of raised intracranial pressure, signs which may be absent. Might, therefore, idiopathic intracranial hypertension be present in some patients diagnosed with chronic fatigue syndrome? Could the two conditions be related?

**Patients and methods:**

From June 2007, patients attending a specialist clinic who fulfilled the diagnostic criteria for chronic fatigue syndrome and in whom headache was an especially prominent symptom were offered CT venography and lumbar puncture, looking for evidence of raised intracranial pressure.

**Results:**

Of the 20 patients who accepted lumbar puncture, eight had pressures of 20 cm H_2_O or greater, including three who had pressures of 25 cm H_2_O or greater. Mean pressure was 19 cm H_2_O.

**Conclusions:**

Some patients with headache and a diagnosis of chronic fatigue syndrome have unrecognised and occult idiopathic intracranial hypertension. The possibility that the two conditions are related cannot be excluded.

## Introduction

Chronic fatigue syndrome (CFS) is a condition of unknown aetiology, characterised by debilitating fatigue and defined by a constellation of symptoms without any accompanying physical signs. It can develop at almost any age and symptoms can last for many years. Treatment is largely supportive. Headache is a common complaint.^1,2,3^

Idiopathic intracranial hypertension (IIH) is a condition characterised by headache and visual disturbance, and it is defined by the presence of raised intracranial pressure but without any discernable cause. It affects mainly young obese women but can develop in either sex, at almost any age, and symptoms can last for many years. Treatment is palliative and largely directed at preserving sight. Though not exciting much comment (because headache and visual symptoms are so consuming), fatigue is a common symptom.^4,5,6,7^

The clinical features of IIH, of which headache and papilloedema are the most common, reflect the abnormal intracranial pressure but are otherwise completely non-specific. Moreover, some patients with IIH may have headache without signs of raised intracranial pressure and these patients can be difficult to diagnose, especially if they do not conform to the usual IIH phenotype of the young, obese female.^6,8^

In order not to miss these cases, therefore, we extended the diagnostic work-up of patients with chronic fatigue to specifically exclude raised intracranial pressure when headache was a prominent symptom.

## Patients and methods

From June 2007 to March 2012, 25 patients attending a specialist clinic, who satisfied the accepted criteria for CFS,^1^ in whom headache was a prominent symptom, were referred for brain CT and CT venography with a view to lumbar puncture. All patients had had symptoms for at least six months. None had papilloedema or any other sign of raised intracranial pressure.

A standard brain CT scan was used to screen for hydrocephalus or intracranial mass. Cerebral CT venography (CTV) was performed at the same time, first, to exclude venous sinus thrombosis and, second, to look for narrowing of the transverse sinuses (now widely recognised as an indicator of raised intracranial pressure).^9,10,11^ If the imaging suggested no contraindication, then patients were offered lumbar puncture; this procedure carried out subsequently in the left lateral position using a 22 gauge needle with pressures referenced to zero at the point of needle insertion.

## Results

25 patients were referred in total. Standard brain CT was normal in all cases. CTV showed no evidence of existing or previous thrombosis in any case. Bilateral focal narrowing of the transverse sinuses was seen in two cases (one subsequently diagnosed with IIH).

20 patients accepted the offer of a lumbar puncture and five refused. Of the 20 who accepted, eight had intracranial pressures of 20 cm H_2_O or greater, including three with pressures of 25 cm H_2_O or greater. Mean pressure in the group was 19 cm H_2_O. The three patients with pressures of 25 cm H_2_O or greater were relabelled as IIH. Of these, two were obese and the other was moderately overweight (see Table 1).

## Discussion

Headache in IIH is generally non-specific. Moreover, IIH can exist without papilloedema or other signs of raised intracranial pressure, meaning, especially in the absence of the usual phenotype, it is easily overlooked as a cause of headache.^8,12,13,14,15^ Mathew and co-workers, for example, used lumbar puncture to screen for IIH without papilloedema in patients with a transformed migraine type of chronic daily headache and found a prevalence of 14% in 85 patients.^12^

Since then, the presence of stenoses in both transverse venous sinuses has come to be recognised as a marker of raised intracranial pressure, a sign which can be detected non invasively.^9,10,11^ Bono et al. screened 724 patients attending an outpatient clinic with migraine for raised intracranial pressure using cerebral MR venography (MRV).^14^ Patients who had bilateral transverse sinus stenoses on MRV were offered lumbar puncture. They found 7% of patients were positive on MRV. Of those who went on to have a lumbar puncture, 70% had raised intracranial pressure; this implying, as a minimum, that 5% of their patients diagnosed with migraine actually had IIH.

The same group, using the same method, published almost identical findings in patients with chronic tension headache.^15^ None of the patients in either study had papilloedema and, apart from a shorter history and a tendency to greater obesity, there was nothing to distinguish patients with IIH from the other patients with headache.

MRV is a useful non-invasive tool for diagnosing raised intracranial pressure but has limited sensitivity. Higgins et al., using a similar protocol to Bono et al. showed previously that bilateral transverse sinus stenoses might be seen in 65% of patients with proven IIH.^10^ Other patients with IIH had less striking venous anomalies or none. Relying on this sign to screen for raised intracranial pressure, therefore, will mean underestimating the number of cases. So, the true prevalence of IIHin patients with migraine, for example, is not likely to be less than 8% and could be nearer the 14% reported by Mathew and colleagues.^12^

If patients with unrecognised IIH comprise a significant proportion of patients attending headache clinics, then they probably also form a significant proportion of other patient populations where chronic headache is a feature, including patients with chronic fatigue. So, with a question over the accuracy of imaging techniques in excluding it, we opted for lumbar puncture as the gold standard measurement of intracranial pressure and used CT and CTV primarily to screen for contraindications to lumbar puncture and to exclude alternative diagnoses. Our results, in which 3 patients out of 20 were found to have raised intracranial pressure, would seem to justify this approach.

Interestingly, five additional patients in our series had CSF pressures that were borderline high, if not actually abnormal by the most stringent criteria. Two of these were obese, three were moderately overweight and all would likely have been labelled as IIH if they had had papilloedema. Patients with IIH share other symptoms with CFS apart from headache and fatigue – dizziness, anxiety, depression, arthralgias, for example – all regarded as non-specific.^7,16,17^ Nevertheless, these cases raise the possibility that there might be some overlap between the two syndromes.

Clearly, this paper describes preliminary work. We present a series of observations on patients with chronic fatigue, which is essentially a survey of intracranial pressure at a fairly random point in their illness when headache was severe. There is no contemporaneous control group. Instead, we have relied on historical controls in the form of accepted normative data for intracranial pressure. This is a deficiency but, having said that, what controls would be appropriate against our patient group? Not patients with chronic fatigue without headache because some patients with IIH have no headache. Not patients with headache but without fatigue because some of these patients also might have IIH. Healthy volunteers perhaps?

Even the issue of what constitutes normal CSF pressure is contentious. For research purposes, defining raised intracranial pressure as 25 cm H_2_O or greater usefully excludes normals from a study group. In clinical practice, however, levels of up to 28 cm H_2_O may be accepted as normal. Yet, the studies that inform this normative data either include patients with headache^18^ or conspicuously fail to exclude them^19^ – a testament to hubris if ever there was one.

Characterisation of headache in this series is poor; something also that could be levelled as criticism. This was deliberate. Headache in IIH is non-specific and can mimic named headache syndromes.^8,12,13,14,15^ So, we saw no point in trying to stratify patients into different headache types. This would normally be considered a handicap – a strategy likely to diminish the chance of producing a statistically useful result. Yet, we still found unequivocal IIH in 15% of our patient group.

Patient selection was also ‘unscientific’. That is, it was at the discretion of one of the authors who was running the clinic (Prof. Lever), on the basis of headache being a prominent feature in their illness. Might there have been something else in the history or clinical examination that was offering a clue to the diagnosis of IIH that would have been picked up by any competent physician? This is difficult to refute except to reiterate that the decision to extend the diagnostic work-up of these particular patients was one of policy, developed in recognition that patients with IIH were probably being missed. Moreover, none of the patients referred for lumbar puncture had any signs of raised intracranial pressure, even in retrospect.

It should be appreciated that this work is not an attempt to establish the prevalence of IIH in patients with CFS. It is an exploration of the possibility that the two conditions might be related or, at least, that IIH is being routinely overlooked. To answer these questions as efficiently as possible we selected patients with chronic fatigue in whom IIH was most likely to be found – that is, patients in whom headache was a prominent symptom. Inevitably, this means that the results may not be more widely applicable. Nevertheless, with a pick-up rate of 40% for IIH by some criteria and, at 19 cm H_2_O, a mean CSF pressure only one point short of the same upper limit of normal, it suggests that the issue would benefit from further study.

Finally, it is worth addressing one particular further comment raised in the review process questioning the value of associating CFS with IIH – two difficult diseases, which are poorly understood. We answer this by saying that while our findings may seem to be little more than exchanging one condition of unknown aetiology for another, there is at least little question that IIH is a disease with an organic basis – something that should raise questions about the nature of CFS. It could also open the door to new treatments.^20,21,22,23,24^

## Conclusion

▪ Some patients with headache and a diagnosis of CFS have unrecognised and occult IIH.
▪ The possibility that the two conditions are related cannot be excluded.

## Figures and Tables

**Table 1:** Characteristics of 20 patients diagnosed with CFS who had lumbar puncture

CSF pressure (cm water)	BMI(kg/m^2^)	BTSS (on CTV)	Sex	Age	Length of history (years)
41	36	yes	f	40	1.5
29	31	no	m	53	6
25	27	no	m	60	10
22	22	no	m	21	3
21	39	no	f	46	2.5
20	27	no	f	22	5
20	30	no	m	39	5
20	36	yes	f	49	11
19	31	no	f	26	13
19	23	no	f	16	0.5
17	18	no	f	20	0.8
17	30	no	f	22	8
17	31	no	m	18	0.8
15	23	no	m	62	15
15	26	no	m	47	0.5
14	20	no	f	23	12
14	21	no	f	27	9
12	23	no	f	47	30
12	27	no	f	22	3
12	22	no	f	41	12

BMI: body mass index (normal, 18.5–25; overweight, 25–30; obese > 30)

BTSS: bilateral transverse sinus stenoses

CSF: cerebrospinal fluid
